# Three-Dimensional Printing of the Epineurium for Peripheral Nerve Repair: A Comprehensive Review of Novel Scaffolds for Nerve Conduits

**DOI:** 10.3390/biomimetics11030196

**Published:** 2026-03-08

**Authors:** Alynah J. Adams, Iulianna C. Taritsa, Kaavian Shariati, Aaron I. Dadzie, Jose A. Foppiani, Maria Jose Escobar-Domingo, Daniela Lee, Angelica Hernandez-Alvarez, Kirsten Schuster, Helen Xun, Samuel J. Lin

**Affiliations:** 1 Division of Plastic and Reconstructive Surgery, Beth Israel Deaconess Medical Center, Harvard Medical School, Boston, MA 02215, USA; ajadams@mcw.edu (A.J.A.);; 2 David Geffen School of Medicine, University of California, Los Angeles, Los Angeles, CA 90095, USA; 3 The University of Texas Rio Grande Valley School of Medicine, Edinburg, TX 78541, USA

**Keywords:** 3D printing, additive manufacturing, epineurium, nerve growth factor, peripheral nerve injury, reconstruction

## Abstract

**Background:** Nerve conduits are used to bridge peripheral nerve defects caused by trauma, iatrogenic injury, or oncologic disruption. Three-dimensional (3D) biomimetic scaffolds for peripheral nerve regeneration have advanced significantly in recent years, driven by improvements in printing technology and neuronal seeding techniques. We report on published designer conduits that can recreate the epineurium, a critical yet challenging-to-manufacture feature of nerve tissue. **Methods:** A medical librarian conducted a literature search for our systematic review on EMBASE, Web of Science, and PUBMED, following PRISMA guidelines, for articles from January 2010 to January 2026 for the systematic review. Descriptive statistical analysis was performed using Microsoft 365 Suite software. The literature review was conducted using keywords and search terms describing the history and development of 3DP nerve guidance conduits published prior to January 2026. **Results:** Our search yielded 273 titles, of which 8 were included after full-text review; these studies used 3D printing to generate nerve conduits for preclinical models. Manual data extraction identified studies reporting successful epineurial recreation. The included scaffold materials were polycaprolactone, poly(l-lactide-co-ε-caprolactone), poly(lactic-co-glycolic acid), acrylate resin, and gelatin methacryloyl. In animal model studies, various terms were used to describe the epineurium outer sheath. Despite this variability in nomenclature, many of these reports indicated successful sciatic functional index (SFI) recovery, favorable g-ratios, good durability, high cell viability, and significant neurite elongation at the time of sacrifice. **Conclusions:** 3DP nerve conduits targeting the epineurium are promising approaches for treating peripheral nerve defects. The constructs promote oriented growth and myelination. Future research on incorporating the epineurium into nerve scaffolds may consider encapsulating NGF to promote more efficient nerve regeneration, standardizing the definition of epineurial recreation, designing mechanical and permeability reporting benchmarks, and evaluating cell strategies using comparable functional and histologic endpoints.

## 1. Introduction

Peripheral nerve injuries (PNI) are increasingly common in medical practice and include up to 3% of trauma patients in the United States and worldwide [1]. Although common, peripheral nerve injuries have proven complex, requiring innovative strategies and extensive interprofessional medical teams to address these severe injuries accordingly. These injuries can rapidly become debilitating and change the course of one’s life, with high morbidity rates and long recovery times [2]. PNIs can occur due to several traumatic injuries, with motor vehicle injuries prevailing as the most common cause, and motorcycle crashes are included as a common close second cause of injury [3]. This review focuses on less common but equally debilitating causes of PNI, including nerve stretch injuries, laceration injuries, compression or ischemic injury, iatrogenic injury, and oncologic disruption [1].

Peripheral nerves have a remarkable capacity for spontaneous regeneration, but this self-repair is hindered by obstacles that prevent complete recovery. One of the most common complications of nerve injuries is the formation of a traumatic neuroma. Traumatic neuromas are disorganized proliferative responses that can develop at the proximal end of a transected peripheral nerve anywhere in the body [4]. Neuromas can be excruciatingly painful and cause both psychological and physical debilitation [5]. With the addition of a nerve guidance conduit, the neuroma could be prevented in the long term. Recently, surgical strategies such as targeted muscle reinnervation (TMR) and regenerative peripheral nerve interfaces (RPNI) have been introduced to treat and prevent painful neuromas [6,7,8].

Understanding the anatomy of peripheral nerves helps envision how disruption can be so debilitating for patients and affect clinical diagnosis, prognosis, and treatment. The peripheral nerve trunk has three layers. It begins with the outermost layer, the epineurium, which consists of several fascicles and blood vessels and is surrounded by a specialized cellular sheath, the perineurium, which contributes to the blood-nerve barrier. This layer surrounds the innermost layer of the collagenous matrix, the endoneurium, which contains individual axonal fibers, either myelinated or unmyelinated [9]. All three layers of nerve tissue must be present to ensure proper development and linear growth, an issue that has limited the performance of nerve guidance conduits (NGCs) to date. The epineurium is the layer that reconstructive surgeons focus on during nerve repair.

**Figure 1:**
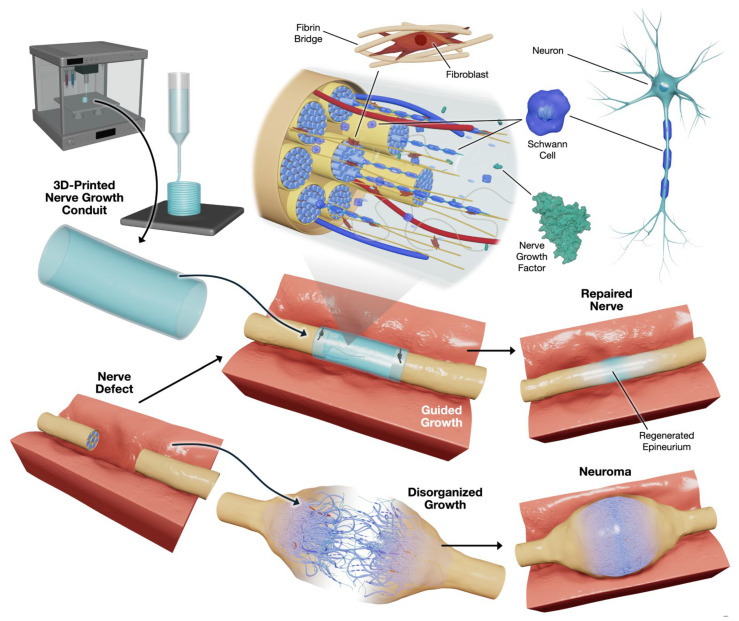
Introduction to 3D printing of nerve guidance conduits to promote linear nerve growth into a regenerated epineurium, thereby avoiding disorganized growth that leads to neuroma formation.

Developing methods to repair traumatic peripheral nerve injuries has long been a focus of interest. These methods include creating natural or synthetic tubular nerve guidance channels as alternatives to autografts, which have shown success [10]. After the realization that nerve guidance channels were the most viable path to long-term, accurate repair of peripheral nerve injury, research began to flourish. In the 20th century, nerve conduits were recognized as the premier method for bridging large peripheral nerve gaps that were not feasible with autografts or allografts [10,11]. The current gold standard for peripheral nerve treatment relies on autografts or autologous grafts, which have numerous limitations, including donor scarcity, donor size mismatch, and various immunological complications [12]. Autografts are excellent for repairing short (<10 mm) nerve deficits, which limits their use in extensive PNI repair. Three-dimensionally printed (3DP) NGCs with integrated cells, improved intraluminal microenvironments, and growth factors may be a promising emerging strategy for addressing a centuries-old medical problem in large-gap PNI repair and neuroma prevention (Figure 1).

Most recently, advances in fabrication techniques have led to a growing focus on the intraluminal microenvironment, which is known to enhance the alignment and organization of neurite outgrowth, thereby enabling better tissue regeneration [13,14,15,16].

This illustration (Figure 1, created by illustrator Kaavian Shariati) depicts the use of 3DP nerve growth conduits to facilitate peripheral nerve repair. The process begins with the fabrication of a specialized conduit using advanced 3D printing techniques, designed to bridge nerve defects. The conduit incorporates critical biological components, including Schwann cells, neurons, fibroblasts, and NGFs. When applied to a nerve defect, the conduit provides structural support and directional cues, guiding regenerating nerve fibers to restore the nerve’s natural architecture, including the epineurium and fascicular structures (Table 1).

Properly guided growth within the conduit promotes organized nerve regeneration and reduces the risk of disorganized growth, which can lead to neuroma formation, chronic pain, or muscle atrophy. Ultimately, this approach aims to facilitate the regeneration of a functional, structurally intact nerve, demonstrating the potential of combining bioengineering and regenerative medicine for improved clinical outcomes in nerve repair (Table 1).

Over the years, three-dimensional printed peripheral nerve guidance conduits have emerged as a promising approach for bridging peripheral nerve defects. As modern technology advances and neuronal seeding techniques improve, new evidence suggests that 3DP designer conduits can recreate the epineurium layer by enhancing tissue regeneration, spatial precision, and localization within the conduit. It is suspected that incorporating the epineurium layer provides a solid foundation for a successful 3DP NGC. Although challenging, this method is important for translating NGCs for surgical procedures, and this review highlights eight studies with promising preliminary results. Understanding the shared qualities of these studies can encourage the continued pursuit of high-quality nerve repair and reinnervation, the avoidance of future neuroma development, and improved patient quality of life.

3D printed nerve guidance conduits (3DP NGCs) are increasingly sophisticated, enabling improved microarchitecture, including patient-matched diameter, length, and geometry, and integrating pro-regenerative cues (e.g., growth factors and strategies to support vascular ingrowth, multilayered composites, and microchannels) in order to match the irregularity of native nerve architecture [17,18,19,20]. However, despite strong preclinical engineering performance, many 3DP NGCs do not translate well to operative use because they do not reliably incorporate a surgically handleable, suture-retentive outer layer analogous to the epineurium, the structure surgeons rely on for secure coaptation and tension-bearing suture purchase in native tissue. It is likely that the functional units of the native nerve, namely the epineurium, perineurium, and endoneurium, provide a microenvironment suitable for optimizing neurite growth and promoting neural regeneration.

This review is therefore framed by clinical priorities: not only whether a conduit promotes axonal regeneration, but also whether it recreates the structural function of the native epineurium at the repair interface, thereby making it suitable for surgical manipulation. A second barrier to translation is inconsistent terminology and incomplete reporting: across the literature, epineurium-like features are variably labeled as an “outer shell,” “sheath,” or “neurium-mimetic” layer, often without quantitative mechanical criteria (e.g., tensile/tear properties or suture pull-out strength) to substantiate epineurium equivalence. Establishing clear reporting standards for an “epineurium-mimetic” layer will improve interpretability, reproducibility, and surgical readiness of 3DP NGCs.

## 2. Methods

### 2.1. Systematic Review Methods

#### 2.1.1. Eligibility Criteria

All studies used 3D printing to produce nerve guidance conduits, which successfully developed the epineurium layer within the NGC. Other inclusion criteria included controlled randomized trials, validation studies, and experimental articles written in English. Editorials, literature reviews, systematic reviews, meta-analyses, case reports, commentary reports, abstracts, and editor letters were excluded from this review. To ensure a comprehensive review, articles were gathered without imposing restrictions on publication year, journal, country of origin, or other limiting parameters. Eligibility criteria were defined based on a selective literature review of the study subject. Articles that met the predefined inclusion and exclusion criteria above were collected for data extraction. The study is registered in PROSPERO (CRD42023473704) [21].

#### 2.1.2. Information Sources

A comprehensive literature search was performed in January 2026 by our team and a medical librarian, following the Preferred Reporting Items for Systematic Reviews and Meta-Analyses Extension for Systematic Reviews (PRISMA) guidelines [22]. The databases searched included MEDLINE, Embase, Web of Science, Cochrane Central Register, and ClinicalTrials.gov.

#### 2.1.3. Systematic Review Search Strategy

An experienced medical librarian designed the search strategy using subject terms, subject headings, and keywords related to surgical education, three-dimensional and 3D printing, printing, additive manufacturing, epineurium, nerve conduits, nerve guidance conduits, and nerve guidance channels. The Covidence system includes a primary screening step after articles are imported to reject duplicates and irrelevant articles [23]. Our team reviews these irrelevant articles for mistakes before continuing our screening and selection process. Once the articles that met our descriptors were collected, the librarian uploaded them to Covidence for our team to screen and review.

#### 2.1.4. Selection Process

The online review program Covidence was used to import the search results [23]. Two independent reviewers conducted a two-stage screening process for study selection. First, subjects were identified by screening article titles and abstracts. Next, the same two reviewers conducted a full-text analysis. All discordances, including grey literature, in either stage of review were resolved by a third researcher, who moderated the discussion until a joint decision was made. Once all articles had been thoroughly evaluated against our eligibility criteria, they were exported into a data extraction table. The protocol for this review was registered on PROSPERO under the identification number CRD42023473704 [21].

#### 2.1.5. Data Collection Process

A structured literature search was conducted across the OVID, EMBASE, and PubMed databases for articles published in. Seven independent reviewers extracted the data from the final articles using predesignated variables. These variables included the first author’s last name, publication year, journal title, type of study (in vivo, in vitro, or mechanical testing), and the total number of samples. Our group also collected print technique data on resolution, base materials used, cells or trophic factors included, the architecture details, animal model, fabrication technique, epineurial collapsibility and flexibility, reproducibility, sciatic functional index (SFI), g-ratio, neurite elongation, and nerve scaffold structural integrity. The collected biomechanical and structural properties encompass scaffold tensile strength, Young’s modulus, durability, cell density, neurite outgrowth speed, print fidelity, and long-term cell viability. Finally, logistical measures were considered, focusing on functional comparisons with the gold-standard technique using the native nerve. Additionally, the barriers, limitations, and challenges associated with each method of nerve conduit production, as well as the potential for rapid prototyping, were evaluated. In cases of unclear information, a second reviewer was consulted for additional insight, and consensus was reached.

#### 2.1.6. Data Items & Outcomes

The primary outcomes sought were creation of an epineurium or outer sheath in a 3DP NGC. Our secondary outcomes included growth factor, epineurial scaffold structural integrity, SFI recovery, g-ratio, durability, cell viability, cell density, neurite outgrowth speed, and maximum elongation. Other variables included author, title, year, publication year, journal, country of publication, instrument used for 3D printing, the fabrication technique, base material, NGC architecture, OHAT and NIH QA ratings, and experimental model.

#### 2.1.7. Statistical Analysis

Descriptive statistics were used to compare the frequent use of specific print settings and bioink utilization. We reported the variables corresponding to the numeric values and mechanical properties of the 3DP scaffolds. Any subgroup analyses were described qualitatively.

#### 2.1.8. Quality Assessment and Risk of Bias Tools

The National Institutes of Health (NIH) Quality Assessment tool [24] and the Office of Health Assessment and Translation (OHAT) risk-of-bias tool [25] were used to assess the quality and potential for bias of the papers undergoing full-text review, respectively. Each study was evaluated using the NIH Quality Assessment tool’s criteria and categorized as ‘good’, ‘fair’, or ‘poor’. One point was awarded for each item included in the study, for a maximum of 9 points. A study was rated ‘good’ (the lowest risk of bias) if ≥7 points were awarded, ‘fair’ (moderate risk of bias) if between 7 and 5 points were awarded, and ‘poor’ (high risk of bias) if ≤4 points were awarded. The studies were evaluated using the OHAT criteria and categorized as having a potential risk of bias of ‘definitely low’, ‘probably low’, ‘probably high’, and ‘ definitely high’.

### 2.2. Literature Review Methods

#### 2.2.1. Literature Review Search Strategy

A comprehensive literature review was performed on 18 January 2026. The search, conducted across PubMed/MEDLINE and Web of Science, included keywords, title headings, and abstracts for all papers published prior to that date.

#### 2.2.2. Study Selection

The comprehensive search included all studies, including editorials, systematic reviews, and original articles describing peripheral nerve conduits that are 3DP or additively manufactured or where printing is core to the architecture, that report at least one of the following: (1) mechanical testing, (2) degradation kinetics, (3) architecture metrics, or (4) in vivo repair outcomes. These studies must also identify their biomaterials and printing modality. Excluded studies include purely electrospun materials not integrated into a 3D printing modality, studies that focus only on central nervous system biomaterials, and studies that do not clearly specify materials or processing modalities.

#### 2.2.3. Data Extraction and Synthesis

A narrative analysis of the included studies was synthesized to describe the use of three-dimensional printing for nerve guidance conduits in plastic, orthopedic, and neurological surgery.

## 3. Results

### 3.1. Systematic Review Results

**Figure 2:**
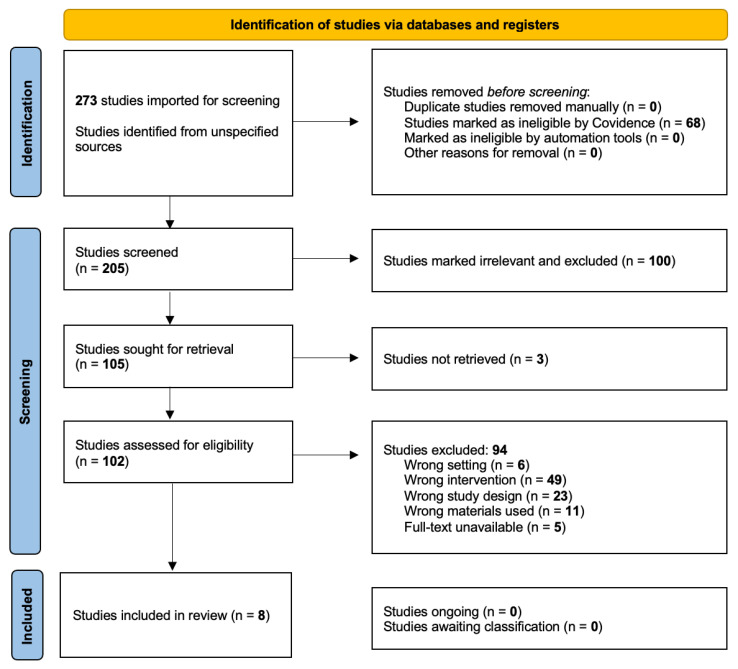
Identification of studies via databases and registers.

Our search identified 273 titles for review; after full-text screening, 8 articles remained, including relevant studies that used 3D printing to generate the epineurial layer in nerve conduits (Figure 2). Across all extracted studies, manual data extraction identified eight unique studies reporting successful epineurial recreation [26,27,28,29,30,31,32,33].

Chen (2020) demonstrated that 3DP GelMa/GC-MSs hydrogels mimicking the epineurium layer supported Schwann cell proliferation and promoted neurite outgrowth, thereby improving the organization of nerve guidance structures [26]. Lee (2022) reported that a 3DP PLCL-gelatin hydrogel NGC with an epineurial-like layer successfully facilitated axonal regeneration [27]. Li (2021) investigated neural crest stem cell-derived (NCSC) Schwann cell progenitors incorporated into 3D-printed NGCs [28]. These conduits used superfine fibers fabricated with high-resolution electrohydrodynamic 3D printing, which facilitated axonal regeneration through mechanisms such as elongation, cell migration, adhesion, and neurite alignment [28]. Rodriguez-Sanchez (2025) created a 3DP PCL NGC containing multi-functionalized canine adipose-tissue-derived mesenchymal stromal cells (AdMSCs) with heterologous fibrin biopolymer (HFB) and a sputter-coated gold outer shell to assess the functional and electrophysiological mobility in rats following nerve injury with the fused filament fabrication (FFF) printing approach [29]. Fang (2023) compared a conductive multiscale tri-layered PCL 3DP microfiber scaffold with epineurium/“shell” made of PCL/collagen nanofibers and innermost layers of reduced graphene oxide(rGO)/PCL microfibers tested against autografts in the rat model (Table 2) [30]. Fan (2025) demonstrated success using electrohydrodynamic jet 3D printing (E-jet) and electrospinning to fabricate PCL 3DP NGC for large-gap nerve deficits in a rat model, compared with the autologous control with an “outer layer” for enhanced strength and prevention of surrounding structure infiltration, and a focus on the composite intraluminal microenvironment [31]. Chang (2025) utilized lithography with a photoinitiator to create an acrylate resin NGC with a laminin outer sheath [32]. The NGC does not contain any cellular enhancements or trophic factors but demonstrates the fabrication of a successful epineurium with a bilayered architecture of micro- and nanofibers [32]. Kong (2024) is the only paper to have mimicked all three layers of native nerve tissue in their SpinMed graft, with their 3DP epineurium sheath performing above native nerve, exhibiting high permeability to nanoscale proteins and low permeability to musculoskeletal-pericytes [33]. The SpinMed is created from PCL, hexafluoroisopropanol (HFIP), and silk fibroin (SF) to optimize the trilayered composite NGC graft in mice, rats, and canine models using phase separation for the epineurial layer and crosslinking for the perineurial NGC layers [33].

Overall, these studies underscore the promising potential of 3DP nerve guidance conduits to facilitate nerve regeneration and prevent neuroma formation by enabling neurites to grow along oriented microfibers or microgrooved architectures in a geometric pattern [28]. Various designs and materials have demonstrated efficacy in preclinical models, with hydrogel networks, such as gelatin-based hydrogels and crosslinked gelatin, showing the most success by effectively mimicking the native epineurium layer (Table 2). While most studies recorded nerve regeneration properties at the date of model sacrifice, it is possible that nerve regeneration would have continued to improve over time. This dataset summarizes the key outcomes of the eight studies evaluating the efficacy of various tissue-engineering approaches for nerve regeneration. The scaffold materials comprised biodegradable polymers, including polycaprolactone [28,29,30,33], poly(l-lactide-co-ε-caprolactone) [27], and poly(lactic-co-glycolic acid) [31], as well as gelatin methacryloyl chitosan [26] and acrylate resin [32].

Chen (2020) incorporated NGF into a gelatin/methacryloyl chitosan microsphere scaffold that maintained structural integrity for 12 weeks and degraded within 3 days, with no signs of graft disconnection or adverse neuroma formation [26]. The 4-layer scaffold showed high cell viability (97.1% ± 3.69%) and supported robust neurite outgrowth, with an average maximum elongation of 14.51 ± 6.86 μm (Table 3). The 3DP shell incorporated NGF, mimicking the epineurium and protecting Schwann cells as they organized into a linear growth pattern [26].

In contrast, Lee (2022) also included NGF with similar structural and durability periods, but demonstrated slightly lower cell viability at 88.7% ± 0.7% [27]. Notably, this study reported a high neurite extension rate of 26.8 ± 0.8 μm per day, achieving an average maximum elongation of 26.8 μm. The bioprinted poly(lactide-co-ε-caprolactone) scaffold was light-crosslinked with a gelatin hydrogel using an LED curing lamp. The scaffold, similar to Chen (2020), maintained integrity for 12 weeks and remained durable for up to 7 days, although the study did not extend beyond this period [26]. Lee reported successful regeneration in the rat model by measuring the myelin thickness, the ankle angle at terminal stance, weight gain, tetanic forces, and muscle weight in place of the sciatic functional index (Table 3) [27].

Li (2021) incorporated neural crest stem cells (NCSCs) into a 3DP polycaprolactone scaffold using the melt electrowiring technique to promote neurite cell growth and elongation and observed increased cellular proliferation over 10 weeks, with improved structural integrity and durability, though specific viability percentages and neurite metrics were not provided [28]. Additionally, they found that this method yielded low mechanical stability due to the small size of MEW-printed fibers. This study highlighted that while the epineurium has a clear role in the organization and growth of a neurite, implanting a 3DP layer of epineurium alone is insufficient to ensure organized, linear regenerated neurite growth (Table 3) [28].

Rodriguez-Sanchez (2025) was able to create a scaffold with a monolayer of extracellular calcium matrix with multipotentiality in vitro after a 21 day incubation period [29]. This scaffold expressed neurotropic factors brain-derived neurotrophic factor (BNDF), glial-derived neurotrophic factor (GDNF), hepatocyte growth factor (HGF), and interleukin-10 (IL-10) following stimulation with interferon gamma (IFN-*γ*). This group focused on reporting results from rat rehabilitation running tests, such as sciatic and tibial functional indices (SFI = −65.12), nerve stimulation speed, expression of BNDF, GDNF, and HGF, as well as histological and electrophysiological outcomes, without reporting the characteristics of nerve regeneration (Table 3). Complications were not reported, but the study suggested the possibility of rat muscle contracture following NGC implantation [29].

Fang (2023) was able to demonstrate impressive mechanical properties, including 96% of compressive recovery after 100 compressive cycles, and 85% of mechanical strength with minimal deformation following elongation (>100%) and strong tensile strength tension compared to controls [30]. Importantly, Fang (2023) demonstrated competency using the structure pull-out tensile test, with a force of 1.7 N and an elongation of 0.5 mg mL^−1^ [30]. The authors recognize that the structural features of the native epineurium are emulated with the nanofibrous design, which is beneficial for the development of highly organized, functional nerve growth and regeneration, and highlight the importance of a porous external shell capable of free nutrient exchange and metabolic waste, with the benefit of surrounding tissue infiltration prevention [30]. The SFI was impressive at −51.5 ± 8.6, with 2-month durability and nerve elongation at 42.5 ± 1.28 µm. The nanofibrous design successfully mimics the structural characteristics of native epineurium, which is vital for promoting highly organized and functional nerve growth. Furthermore, the design emphasizes a porous external shell, which is important for enabling free exchange of nutrients and metabolic waste while preventing infiltration by surrounding tissue. 

Fan (2025) describes the production of a “dense outer-layer structure” in a successful NGC to support aligned neurite growth and mitigate target muscle atrophy similar to autologous transplantation [31]. The scaffold was created with the base material PLGA via layer-dependent electro-hydrodynamic jet (E-jet) and electrospinning. Several trophic factors were used, including, umbilical cord mesenchymal stem cells (UMSCs). Vertical and horizontal cross-lamination was important intraluminal architecture for the use of this scaffold in the rat model. The structural integrity was intact at rat sacrifice week 12, with a SFI of approximately 62, a g-ratio of approximately 0.60, and the scaffold was durable over 7 days, with 65.6% of cells viable at this time. The neurite grew at a rate of 33.28% with a maximum elongation of 134 µm [31].

Chang (2025) demonstrated comparable functional recovery results between a topographical NGC focused on the epineurium and perineurium layers and a hollow cylindrical NCG in the rat model [32]. This pilot study design did not include growth factors or composite intraluminal microenvironment structures. At 6 weeks, the SFI was reported at −73.24 with a g-ratio of approximately 0.72. The NGC was durable for over 7 days, while the viable cells were over 65% of the control. The neurite grew at a rate 38% faster than the control, reaching a maximum neurite elongation of 134 µm. Notably, the lumen diameters were below the standard (1.0 mm), and the rats were sacrificed at 6 weeks, well before the 12-week standard for nerve regeneration, which limited the reliability of the sciatic functional index (SFI) tests [32].

Kong (2024) developed a trilayered neurium-mimetic composite scaffold to directly emulate the native tissue layers [33]. No growth factors were included in the SpinMed graft design, but the graft was mechanically tested in vitro prior to implementation in the mouse, rat, and canine models. In the rat model, the SFI was approximately −45, the g-ratio was around 0.55, and 50% degradation was reached at 84 days post-implantation. Cell viability, neurite growth speed, and neurite maximum elongation were not reported [33].

Overall, incorporating growth factor and an epineurial layer appears to enhance neurite growth rate and cell viability, suggesting their essential role in improving nerve regeneration conduits. The durability of the NGC in the models may have extended past the date of sacrifice, but data is limited due to time constraints. The NGC’s durability in the models might have lasted beyond the sacrifice date, but this is uncertain due to limited available data. Microvascular capillary density, a factor in maintaining the intraluminal microenvironment, was reported in studies by Fang (2023), Fan (2025), and Kong (2024) [30,31,33]. They established angiogenic capacity by identifying relevant genes through Western blot analysis.

The methodological quality of the included basic science studies was evaluated by the authors using the OHAT Risk of Bias Tool (2015), modified for experimental animal and in vitro studies [25]. The domains assessed included selection, confounding, performance, detection, attrition, and reporting bias (Table 2) [25]. Overall, the risk of bias among the studies varied from definitely low to probably high. The research conducted by Chen (2020) [26], Rodriguez-Sanchez (2025) [29], Fang (2023) [30], and Chang (2025) [32] showed the highest risk of bias, mainly due to the lack of randomization and blinding in the cell culture, imaging analyses, and a lack of robust outcome reporting. The animal research conducted by Lee (2022) [27] was assessed as having a probably low risk, indicating strong experimental control and full follow-up, albeit with limited information on blinding and on the justification of the sample size. Conversely, the studies by Li (2021) [28], Fan (2025) [31], and Kong (2024) [33] demonstrated the lowest risk. This was due to their clearly defined randomization protocols, established control groups, and comprehensive outcome reporting, despite reporting on variables different from those deemed important in this manuscript. Across all studies, detection and reporting bias were generally minimal due to the employment of objective histomorphometric or electrophysiological endpoints and detailed presentation of data. Altogether, these results indicate that, while the included studies were methodologically sound, the limited details on randomization and blinding procedures create some uncertainty about internal validity. The National Institutes of Health Quality Assessment Tool demonstrated excellent quality throughout the studies, with 7 [26,27,28,29,30,31,33] rated ‘good’ and one study, Chang (2025) [32], rated ‘fair’ [24].

### 3.2. Literature Review Results

#### 3.2.1. First Generation Nerve Guidance Conduits

Peripheral nerve repair has been of great interest since before the terms we use to describe nerve injury were widely used [34]. Artico et al. described the peripheral nerve repair contributions of Gabriele Ferrara, which was published in 1543 [35]. Ferrara recognized that suturing the ends of a transected nerve provided a direct pathway to repair [35]. It was not until 1895 that Carl Huber demonstrated viable methods to ensure the success of the sutured ends of a transected nerve, especially with extensive nerve loss [36]. These methods included stretching the nerve, transposition, proximal or distal dissection to gain length, and the introduction of foreign materials, which we recognize today as conduits [37]. In 1942, nerve repair was gaining favorable acknowledgement and developing into a surgical subset of expertise when F. K. Sanders categorized the repair of nerve into two approaches: (1) circumferential manipulation to improve approximation of the transected nerve ends, and (2) introducing biomaterials to bridge the two ends [37]. By the 20th century, Miyamoto demonstrated that peripheral nerve repair under increased tension resulted in poorer outcomes, and then in the 21st century, Hanno Mellessi demonstrated the superiority of nerve autografting techniques over epineural suturing under tension [11,38]. Bridging techniques, particularly with nerve autografts, became the preferred approach, along with the development of further techniques to repair transected nerves under lessened tension [39,40]. Concerns about neuroma development, donor site morbidity, the nerve length and circumference size mismatches, and the limited availability of autografts have led to a search for improved designs that address these issues [41,42,43].

The first generation of synthetic nerve guidance conduits was attempted by Lundborg et al. in 1982 [44]. These NGC were nonresorbable silicone hollow tubes that frequently led to compression syndrome and subsequent removal surgeries [44]. Designs have since improved, and FDA-approved clinical NGCs are now created using appropriate biomaterials with varying success in clinical trials.

#### 3.2.2. Biomechanics and Biomaterials of 3DP NGC

Typically, 3DP NGCs are evaluated based on their ability to resist compression, retain sutures, maintain sufficient flexibility to mobilize around muscles and joints, and maintain strength (Young’s modulus and tensile strength) despite potential swelling or dehydration [45]. The ideal nerve repair device encloses the ability to increase the (1) number, (2) speed, and (3) length of regenerating axons by mitigating the duration of Wallerian degeneration occurring inside the conduit, between the transected nerve ends [45,46].

Three-dimensional nerve guidance conduits are an optimal solution for peripheral nerve repair, especially when nerve autografts are scarce [10,38]. The effectiveness of these bioprinted NGCs hinges on balancing strong biomechanical integrity with nerve growth guidance [47]. The success of NGC repair also depends on the gap length they span (less than 30 mm), biomaterials used, biomechanical reinforcements for structural support, immunomodulatory support, and even the incorporation of NGFs [27,28], embryonic stem cells [48], adipose-derived stem cells [49,50], bone marrow stem cells [51], and others [52] for nerve regeneration [53,54,55]. Some conduits are specifically designed to accelerate regeneration, promote angiogenesis, modulate immune responses, and influence Schwann cell behavior through autocrine mechanisms, especially compared with FDA-approved or clinical standards such as autografts and allografts [45,54,55].

#### 3.2.3. Evolution of Nerve Guidance Conduits

As peripheral nerve repair has historically relied on autologous nerve grafts, the limitations of this approach have become insurmountable for the hundreds of thousands of individuals worldwide who experience peripheral nerve injury requiring repair. These resource constraints encouraged innovation in nerve guidance conduits as allograft substitutes for short-gap repairs. Fabrication of NGC biomaterial over the last four decades has gradually progressed from hollow silicone tubing to later introductions of biodegradable collagen, PGA (poly(glycolic acid)), and PLGA (poly(lactic-co-Glycolic acid)) polymers with the goal of isolating the regenerating nerve from the surrounding traumatized tissue and providing protected guidance for axonal extension between transected ends. This stage of NGC development was limited by a lack of internal architecture and mechanical reliability, prompting the design of a second-generation conduit.

To improve conduit guidance channels, researchers began introducing intricate internal microstructures to promote nerve fascicular anatomy (all three layers of nerve structure), porous lumens, and longitudinally aligned microchannels [13,27,56]. The inclusion of these design enhancements aided axonal alignment. Facilitating the fabrication of these methods proved difficult, limiting reproducibility and scalability due to strict geometric constraints. These constraints were addressed in subsequent designs. Minor changes in channel size or pore orientation introduced new manufacturing workflows and design improvements.

The third-generation NGCs achieved biomaterial and biomechanical optimization by incorporating natural polymers (collagen [14,30], chitosan [13,26,57], gelatin [26,58,59,60], and silk fibroin [61,62]) that combine strength and bioactivity, as well as growth factors, various stem cells, and conductive elements.

Design constraints can be summarized as efforts to recreate native neural tissue, including the ability to generate, receive, and transmit electrochemical signals, achieved through precisely controlled printing parameters that are highly lab- and platform-dependent (Table 1). Intraluminal designs are extremely sensitive to the complexities of crosslinking and the potential for post-print swelling. While Lee et al. used high-concentration gelatin to maintain print fidelity, it caused needle clogging above 25% (*w*/*v*), and excessive swelling could disrupt the internal architecture of the microgrooves [27]. Reproducibility is also affected by the geometry of the microfluidic chip, according to Chen (2020) [26], who reports that it requires specific flow-rate adjustments to produce monodisperse microspheres. Particular focus is given to extrusion pressure and speed ranges that optimize continuous flow while preventing nozzle clogging, thereby defining strand and pore sizes. Many composite NGCs have been successfully produced using multiple fabrication techniques simultaneously. Fang (2023) [30] employed both electrospinning and MEW, while Fan (2025) [31] developed their NGC with E-jet and electrospinning. Chang (2025) [32] used lithography combined with a photoinitiator to create an NGC.

High-resolution strategies are more difficult to reproduce. Li (2021) [28] emphasizes the importance of attaining optimal porosity or surface area without relying on custom electrohydrodynamic printing, using specific parameters such as temperature, voltage, pressure, nozzle distance, and speed. These settings are crucial for producing proper microscale fibers and controlled spacing, which are challenging to replicate without highly specialized equipment and extensive local re-optimization [28]. Each article approaches the printing method differently, which explains why cross-study replication is challenging when small variations in swelling kinetics, microsphere uniformity, and printing resolution affect scaffold success. 3DP NGCs cannot fully predict clinical outcomes, but investing in the living epineurial tube and its microarchitecture can help overcome obstacles such as large nerve defects, chronic denervation, allograft size mismatch, and donor scarcity.

#### 3.2.4. Innovations and Future Direction

Future strategies for NGCs are advancing through the combination of 4D printing, advanced biomaterials, and body-responsive designs [63]. 4D printing enables researchers to fabricate conduits from smart materials that change shape in response to stimuli [63,64]. With this approach, flat, minimally invasive devices can be rolled from flat sheets into tubular structures within the body, attaching without stitches and remaining in place at nerve ends once exposed to body temperature or moisture. Shape-changing hydrogels can rapidly form around cut nerve ends, reducing the need for complex microsurgical repair and improving device-tissue integration.

At the same time, new NGCs are being designed to incorporate electrical and bioactive features that more closely mimic the natural nerve environment. Adding conductive nanomaterials, such as MXene nanosheets and specialized carbon nanotubes, provides these 4D-printed conduits with sufficient conductivity to guide Schwann cell migration and axon growth, and enables real-time monitoring of nerve repair [63,65]. Looking ahead, future designs may include or enhance custom-made conduits based on patient scans [66], complex channel structures optimized by computer modeling [27], and new materials that guide axons by incorporating more native nerve features, including nerve layers [31,32,33]. There is also novel interest in 5D and 6D printing, which would add more advanced timing and biological features [67]. Together, these advances could help NGCs perform as well as, or better than, nerve grafts, while avoiding problems such as donor-site injury and limited nerve supply. Whichever direction the future of biomaterials takes, the role of epineurial recreation, mechanical and permeability benchmark reporting, and growth factor reporting should be standardized using comparable functional and histologic endpoints for viable recreation.

## 4. Discussion

Peripheral nerve injuries occur relatively often and are one of the most significant causes of morbidity for trauma patients across the world. Despite their regular occurrence, they remain a complex injury that has required extensive innovation and technical skill to treat, particularly in the setting of traumatic neuromas. There is a path to long-term, accurate repair of peripheral nerve injuries, and it begins with the discovery of 3DP nerve guidance conduits that include NGFs and the epineurium.

### 4.1. Innovations Driving the Surge in 3DP Nerve Conduit Research

Before 3D printing was established as the method with the greatest potential and advantages, numerous fabrication methods and technologies were developed for preparing nerve guidance conduits, including gas foaming, freeze-drying, melt molding, solvent casting, electrospinning, and phase separation. The rise in 3DP nerve conduit research before 2018 was driven by advances in 3D printing technology and biomaterials. These improvements in 3DP resolution and precision, particularly in techniques such as stereolithography (SLA) and digital light processing (DLP), enabled the creation of more intricate, biologically relevant conduit structures, including those with complex internal geometries that mimic natural nerve tissue. Concurrently, the development and accessibility of biocompatible and biodegradable polymers, such as polylactic acid (PLA), polycaprolactone (PCL), and poly(lactic-co-glycolic acid) (PLGA), provided suitable materials for implantation, further accelerating research and development in this field. These combined advancements enabled the fabrication of more complex and customized nerve conduits, ultimately driving a surge in research before 2018 [68]. A paper published in 2020 examines various 3D printing methods (inkjet, extrusion-based, and light-assisted) and compares their capabilities in terms of printing speed, resolution, and the types of biomaterials they can process [69]. The review then analyzes a range of biomaterials, including hydrogels (both natural and synthetic) and decellularized extracellular matrix, and assesses their suitability for bioprinting based on factors such as biocompatibility and printability [69]. We believe the primary purpose of nerve guidance conduit printing is to prevent neuroma development while promoting neurite proliferation, organization, and improved elongation. This review encourages continued research in clinical environments and the standardized reporting of conduit components to ensure reproducibility.

### 4.2. Successful Printing of Epineurium Layer, a Major Advancement

With advancements in modern technology and enhanced neuronal seeding techniques, new evidence supports the development of individualized, designer conduits that can replicate the epineurium layer for patients through bioimaging and biomanufacturing technologies. This technique avoids the difficulties associated with nerve autografts, which include loss of donor function, neuroma formation, nerve distortion or dislocation, and common nerve diameter mismatch. 3DP NGCs can simulate the structure and function of peripheral nerves with various biomaterials, biomolecules, and cells (Table 3) [33,70].

The epineurium’s 3DP properties, including biocompatibility, biodegradability, suitable mechanical properties, and permeability, make it advantageous for creating 3DP NGCs that facilitate axonal regeneration across nerve gaps. The conduit’s architecture can be further optimized to enhance its performance (e.g., by incorporating filaments, sponges, or multiple channels) and support better functional outcomes for long-term patient use. Incorporating Schwann cells or neuronal growth factors into the NGC further enhances nerve regeneration and improves strength and compatibility with the NGC recipient (Figure 1) [71].

Although the inclusion of the epineurial layer significantly benefits the overall success of NGCs, Li et al. found that the epineurial layer alone leads to poor nerve regeneration [28]. This results in the formation of thin, dispersed myelinated and non-myelinated nerve fibers. In contrast, a 3DP scaffold that incorporates both the epineurium and Schwann cell progenitors supports improved nerve regeneration compared with using either the epineurium or Schwann cell progenitors alone. This improvement is evident in the work of Kong et al., in which the development of all three neurium-mimetic layers results in improved outcomes in both rat and canine models. These models exhibit the lowest reported SFI and the greatest durability [33]. However, it is challenging to draw definitive conclusions because each study reports different time points for animal sacrifice, ranging from 6 to 12 weeks. It is assumed that the longer time points allow for more significant nerve regeneration. While the epineurium enhances the conduit, it is not effective for sole autonomic nerve regeneration [28].

Alternative designs to epineurial regeneration strategies include multilevel mimetic scaffolds, aligned core–shell scaffolds, multiscale composite scaffolds, and functionalized NGCs [29,31,33,72,73,74]. While these alternatives are promising, they often fail to fully replicate the native nerve’s hierarchical structure, mechanical protection, and regenerative microenvironment, which evidence suggests could be important for optimal functional recovery [75,76,77,78].

As the successes of 3DP become more apparent, there is more to discover in emerging 4D printing techniques. This technology is regarded as the further development of 3DP, with time as the 4th dimension, along with hydration, temperature, pH, light stimulation, electrical stimulation, and other characteristics of smart materials [79,80]. Tibbits et al. characterized the fourth dimension as the programmed ability of materials to self-transform and modulate cellular activity in response to post-fabrication stimuli [81]. Current 4D printing constructs are known to facilitate the gradual degradation of 3DP scaffolds and the maturation of 3DP tissues due to their time dependence [80,82,83]. The advent of 4D printing technology in tissue engineering and regenerative medicine represents a paradigm shift, offering unprecedented opportunities for bioinks, biomaterials, and biomedical frameworks to address long-standing medical challenges such as PNI [84,85].

### 4.3. Nerve Growth Factor: A Promising Enhancer for Aligning Schwann Cells and Neurons

NGF is an important neurotrophic factor that can provide a beneficial microenvironment to promote nerve regeneration. NGF delivered via conduits may significantly enhance morphological and/or functional recovery of transected and repaired nerves. Evidence highlighting NGF’s role includes its capacity to promote blood vessel growth, increase myelination, and improve conduction velocities compared with externally added Schwann cells. Another study by Yildiz found similar results: NGF is considered ideal for enhancing nerve regeneration due to its role in promoting Schwann cell proliferation and improving recovery outcomes, as evidenced by comparisons with glial growth factor in nerve repair [86].

This notion was supported by Liu et al., who found that combining NGF microspheres with chitosan conduits significantly improved facial nerve regeneration compared with NGF alone or saline [87]. Chen (2020) and Lee (2022) reported similar results [26,27]. Additional trophic factors have shown similar rates of success, including NCSC, AdMSCs, and UMSCs. More research is needed to compare the success of NGCs that express growth or trophic factors. The improvement was evident from reduced muscular atrophy, increased nerve conduction velocity and amplitude, and enhanced histological features, including increased nerve fiber diameter, number, alignment, and myelin sheath thickness [87]. Although reduced muscle atrophy, determined by myelination thickness, muscle wet weight, ankle angle at terminal stance, and contralateral comparison, was acknowledged by included studies.

### 4.4. Challenges with the Development and Application of 3DP NGCs

Incredible strides have been made in discovering and utilizing 3DP NGCs, but significant limitations still remain in their low-cost, mass production. Material challenges include selecting biocompatible and biodegradable polymers with optimal mechanical properties (strength, flexibility, degradation rate), achieving appropriate porosity and permeability for nutrient transport while preventing unwanted tissue ingrowth, and ensuring cost-effective, scalable production with reliable sterilization methods [68,69]. Manufacturing limitations include accurately replicating the complex architecture of nerve tissue, precisely controlling micro- and macro-scale structural features during printing, and reliably incorporating cells and growth factors while maintaining their viability and controlled release [69]. Yarali et al. discuss mechanobiology from the perspective of drug delivery systems and the intricacies of high precision in this context [88]. This underscores the inherent challenges of scaling 3D constructs for practical, clinically relevant applications. Furthermore, achieving consistent and reproducible results across multiple batches is essential for clinical translation [7]. Clinically, the effectiveness of current NGCs for bridging large nerve gaps remains limited, and ensuring seamless integration with host tissue to avoid immune reactions is crucial [70,71]. Next-generation multimaterial tissue-engineered products successfully construct microenvironments that readily facilitate cell activity and tissue regeneration in response to external stimuli [89]. These microenvironments are the closest new technologies have come to appropriately mimicking the native physiological properties of the human body. Finally, the lengthy clinical trial and regulatory approval processes further complicate the widespread adoption of 3DP NGCs [90,91,92].

### 4.5. Limitations and Future Directions

To address current limitations, future research should focus on developing novel biomaterials, advancing 3D printing techniques to achieve higher precision and reproducibility, and optimizing strategies for integrating cells and growth factors [66,93]. Extensive preclinical and clinical testing is essential to demonstrate safety and efficacy. Incorporating cells and neural growth factors into 3DP conduits has proven crucial for tissue engineering, as it enhances tissue regeneration and ensures precise spatial localization within the conduit [18,94]. Mimicking the native nerve architecture, such as the epineurium and fascicular-like guiding structures, can significantly improve the functional performance of nerve conduits [26,33]. Without a consistent naming structure, it is difficult to ascertain the successful viability of the 3DP NGC for clinical use. Overall, integrating the epineurial construct aligns with the broader goal of refining 3D printing methods, improving cell and growth factor incorporation, and enhancing clinical applicability to optimize patient outcomes.

Despite recent advances in biomaterial development, persistent challenges remain in creating epineurium-mimetic constructs that enable high-quality clinical and translational research. Although this comprehensive review includes only 4 studies and heterogeneity precludes meaningful quantitative analyses, we still identified important conclusions that advance the field. The epineurium reconstruction was inconsistently described, with widely variable outcomes, limiting our ability to compare the methods effectively. A major obstacle is the limited reproducibility and reliability of preclinical studies, which often have small sample sizes, inadequate controls, and weak statistical methods that do not accurately predict clinical results. Scaling up for translational use will face challenges related to regulatory approval, sterilization, and ensuring consistent batch quality. Critical clinical endpoints, such as electrophysiology and functional recovery, as well as histology and cell viability, will pose substantial hurdles to the practical application of these methods. These results can be challenging to interpret appropriately and apply to real-world patient scenarios.

## 5. Conclusions

Three-dimensional printing has rapidly transformed the landscape of peripheral nerve repair, offering a promising path toward more precise, biologically informed, and customizable nerve guidance conduits. Recent advances in printing technologies, biomaterials, and biofabrication strategies have enabled the development of increasingly sophisticated conduits that mimic the structural and functional complexity of native nerves. These innovations not only improve regenerative outcomes but also expand the educational utility of NGCs by providing realistic, patient-specific training models.

In sum, 3DP nerve conduits, particularly those that include the epineurium, are promising avenues for treating peripheral nerve defects resulting from various traumatic events. The constructs promote oriented growth and myelination to prevent neuroma formation. Future research on incorporating the epineurium into nerve scaffolds may consider encapsulating NGF or NCSCs to promote more efficient nerve regeneration and organized growth. Continued innovation in epineurial and fascicular–mimetic architectures, alongside improved study design and clinical testing, will help bridge the gap between experimental promise and therapeutic reality. Ultimately, overcoming these challenges will position 3DP nerve guidance conduits as a transformative option for restoring nerve function and improving patient outcomes in the treatment of peripheral nerve injuries.

## Figures and Tables

**Table 1:** Design, engineering, and additive manufacturing considerations for three-dimensional printed nerve guidance conduits.

Category	Consideration	Rationale	3D-Printing/Manufacturing Notes
Conduit Design and Architecture			
Clinical	Clinical Indication/Gap Length	Conduits are most reliable for small-to-moderate gaps; larger gaps often require grafting or augmented designs.	Stratify outcomes by defect length; ensure printed constructs are intended for the target gap range.
Geometry	Inner Diameter Matching	Match conduit inner diameter (ID) to nerve outer diameter (OD) to reduce mismatch, stump constriction, and dead space.	Use patient-/specimen-specific CAD; confirm ID after swelling and post-sterilization.
Mechanical	Wall Thickness/Anti-Collapse	Adequate hoop strength prevents lumen collapse and kinking while preserving flexibility for placement.	Tune wall thickness/infill to balance stiffness and flexibility; test under bending and compression.
Handling	Suture Retention/Handling	Suture retention strength and handling determine the feasibility of atraumatic microsurgical fixation.	Consider reinforcement/anisotropy to increase retention without excessive stiffness; report retention metrics.
Microarchitecture	Porosity And Permeability	Porosity must permit diffusion of nutrients/waste while limiting fibrous tissue invasion and maintaining guidance.	Programmed porosity gradients are feasible with AM; quantify porosity/permeability and relate to outcomes.
Microarchitecture	Intraluminal Microchannels/Multi-lumen	Aligned channels reduce axonal dispersion and provide contact guidance compared with hollow tubes.	Channel diameter/spacing should reflect printer resolution; validate channel patency post-fabrication.
Microarchitecture	Topographical Guidance (Grooves/Fibers)	Microscale anisotropy and surface features guide Schwann cell alignment and axonal extension.	Incorporate microgrooves via high-resolution printing or templating; quantify feature fidelity.
Intraluminal cues	Hydrogel/ECM Lumen Fillers	Fibrin/collagen matrices can stabilize the regeneration pathway and support cell migration within conduits.	Hybrid constructs (rigid shell + soft filler) can be enabled by multi-material printing/assembly.
Biochemical cues	Neurotrophic Factor Delivery	Sustained, localized delivery of NGF/GDNF can enhance neurite outgrowth and regeneration.	Design affinity-/carrier-based release and quantify kinetics after full manufacturing + sterilization workflow.
Cellular cues	Cell Seeding (Schwann Cells)	Support cells provide trophic and myelination cues, particularly valuable for longer gaps and complex injuries.	Bioprinting or post-seeding must preserve viability and phenotype; report cell retention/distribution.
Bio-functionalization	Immuno-modulation/Fibrosis Control	Minimize chronic inflammation and perineural scarring; favor pro-regenerative immune responses.	Surface functionalization and bioactive coatings can be printed or post-modified; include immune/fibrosis endpoints.
Bioelectrical	Electro-conductivity/Electrical Stimulation	Conductive conduits and/or electrical stimulation can accelerate axonal regeneration and functional recovery in models.	Conductive fillers can alter printability and degradation; control conductivity spatially and temporally.
Material and Engineering Considerations			
Biocompatibility	Biocompatibility	Materials must be non-cytotoxic and support Schwann cell adhesion; residual solvents/photoinitiators can be problematic.	For SLA/DLP, optimize washing/post-curing and verify extractables; report cytotoxicity per ISO-relevant assays when possible.
Mechanical	Mechanical Compliance Matching	Match stiffness to native nerve to reduce stress shielding and micromotion-associated fibrosis.	Tune modulus via polymer selection, infill, and microarchitecture; report compressive/bending properties.
Degradation	Degradation Profile	Degradation should maintain lumen patency through early healing and resorb as function recovers.	Porosity/crystallinity (affected by printing) and polymer chemistry influences degradation; track mass loss and mechanical retention over time.
Barrier function	Barrier To External Cell Invasion	Prevent infiltration of fibroblasts and scar tissue while allowing diffusion across the wall.	Use multilayer constructs (dense outer + porous inner) enabled by multi-material printing/assembly.
Translational	Sterilizability/Regulatory Compatibility	Materials and architectures must tolerate sterilization without deformation or loss of properties.	Test steam/EtO/VHP/gamma effects on dimensions, mechanics, and surface chemistry for each print material.
Additive Manufacturing and Process Considerations			
Process	Printing Modality Selection	Printing process governs resolution, achievable porosity, and compatible biomaterials (thermoplastics vs. hydrogels/resins).	FDM/DIW enable robust thermoplastics; SLA/DLP offer higher resolution; bioprinting supports cell-laden inks.
Quality Control	Dimensional Accuracy/Validation	Dimensional fidelity is essential for nerve size matching and channel patency.	Use µCT/optical metrology to quantify deviations pre/post sterilization and after swelling.
Bioink/material	Rheology/Printability	Viscosity, shear-thinning, and gelation kinetics govern strut fidelity and (for bioprinting) cell survival.	Report rheology, nozzle diameter, extrusion pressure, and temperature; link to feature resolution and cell viability.
Chemistry	Crosslinking/Curing	Crosslinking must provide stability while maintaining cytocompatibility and minimizing toxic residues.	Optimize photoinitiator type/concentration and UV dose; implement validated wash/post-cure protocols.
Post-processing	Post-Processing/Sterilization Effects	Post-processing can change surface chemistry/mechanics; sterilization can introduce shrinkage or warping.	Characterize mechanical properties and cytocompatibility after the complete workflow (print, post-cure, sterilize).
Quality systems	Reproducibility/Batch-To-Batch Control	Translation requires reproducible architecture and properties across prints and batches.	Define acceptance criteria (porosity, modulus, ID) and include process controls and reporting standards.

Abbreviations: ID, inner diameter; OD, outer diameter; ES, electrical stimulation; FDM, fused deposition modeling; DIW, direct ink writing; SLA, stereolithography; DLP, digital light processing; EtO, ethylene oxide; VHP, vaporized hydrogen peroxide.

**Table 2:** Summary of key studies evaluating 3DP NGC materials, architecture, and assessment tools.

Author, Year	3DP Instrument and Manufacturer	Fabrication Technique	Base Material	Cells or Trophic Factors Used	Architecture	OHAT Rating	NIH QA	Model
Chen, 2020 [26]	EnvisionTec: 3D-Bioplotter Developer Series, 3DSMAN, NJ, USA	Prepared via microfluid chip	GC-MSs	PC12 & RSC96; NGF	Multiscale composite scaffold; Epineurium layer	Probably High	Good	In vitro
Lee, 2022 [27]	Dr. Invivo ROKIT, South Korea	Light-crosslinking	PLCL	NGF	Microgrooves; Non-Collapsible epineurium	Probably Low	Good	Rat
Li, 2021 [28]	EFL-MDW5800, Suzhou Intelligent Manufacturing Research Institute, SuZhou, China	MEW	PCL	NCSC	Multi-scale scaffold; superfine fibers *, inducing effects; endogenous tissue envelope	Definitely Low	Good	Rat
Rodriguez-Sanchez, 2025 [29]	FAB@CTI, Renato Archer Information Technology Center, São Paulo, Brazil	FFF	PCL-HFB	AdMSCs	Sputter-coated gold exterior	Probably High	Good	Rat
Fang, 2023 [30]	NR	Electrospinning; MEW	PCL, rGO, Collagen	PC12; RSC96	Multi-scale (trilayered) with nanofibers and microfibers	Probably Low	Good	Rats
Fan, 2025 [31]	NR	E-jet; Electrospinning	PLGA	UMSCs; dECM; PC12, RSC96	Bilayered; EVs with vertical & horizontal cross-lamination	Definitely Low	Good	Rats
Chang, 2025 [32]	Photonic Professional GT2 3D printer Nanoscribe, Karlsruhe, Germany	Lithography with photoinitiator	Acrylate resin with laminin	None	Bilayered; micro- and nano-fibers	Probably High	Fair	Rats
Kong, 2024 [33]	BioScaffolder 4.2, GeSim, Radeberg Germany	Phase separation; Cross-linking	PCL, Collagen, & SF	dnECM; mSCs; RSC96	Trilayered; hydrophobic with 1 µm pores	Definitely Low	Good	Mice; Rat; Canine

OHAT Rating = Office of Health Assessment and Translation Rating; NIH QA = National Institutes of Health Quality Assessment Tool rating; MEW = melt electrowriting; E-Jet = electro-hydrodynamic jet; FFF = Fused Filament Fabrication; GC-MSs = Gelatin methacryloyl/Chitosan Microspheres; PLCL = poly(lactide-co-ε-caprolactone); PCL = Polycaprolactone; HFB = heterologous fibrin biopolymer; rGO = reduced graphene oxide; PLGA = poly(lactic-co-glycolic acid); SF = silk fibroin; NGC = nerve guidance conduits; 3DP = three-dimensional printed; NGF = Nerve Growth Factor, NCSC = neural crest stem cells; AdMSCs = adipose-tissue-derived mesenchymal stromal cells; UMSCs = umbilical cord mesenchymal stem cells; dECM = decellularized extracellular matrix; dnECM = decellularized nerve extracellular matrix; EVs = extracellular vesicles; * Superfine fibers are a similar diameter to that of a cell (μm).

**Table 3:** Characteristics of nerve regeneration based on epineurium creation (*n* = 8).

Author, Year, (# of Samples)	Growth or Trophic Factors Embedded	Structural Integrity (Weeks)	SFI Recovery	g-Ratio	Durability (Days)	Cell Viability **	Neurite Outgrowth Speed ***	Neurite Maximum Elongation (µm)
Chen, 2020, (9) [26]	NGF	12	N/A	NR	<3	96.9 ± 1.52%	Robust	14.51 ± 6.86
Lee, 2022, (3) [27]	NGF	12	NR	NR	<7	88.7 ± 0.7%	26.8 ± 0.8	26.8 ± 0.8
Li, 2021, (20) [28]	NCSC	10	−73.72 ± 1.398	~0.84	70	Increased	NR	NR
Rodriguez-Sanchez, 2025 (5) [29]	AdMSCs	NR	−65.12	NR	NR	NR	NR	NR
Fang, 2023 (12) [30]	No	8	−51.5 ± 8.6	NR	60	NR	~38%	42.5 ± 12.8
Fan, 2025 (6) [31]	UMSCs	12	~−62	~0.60	>7	65.6%	33.28%	134
Chang, 2025 (6) [32]	No	6	−73.24	~0.72	NR	NR	NR	NR
Kong, 2024 ^‡^ (5) [33]	No	12	~−45 *	~0.55 *	84 *	NR	NR	NR

# = number; ^‡^ Estimated values for this study; NGF = nerve growth factor; NCSC = neural crest stem cell; AdMSCs = adipose-tissue-derived multipotent mesenchymal stromal cells; UMSCs = umbilical cord mesenchymal stem cells; NR = not reported; N/A = Not applicable; * Rat model; ** Reported as *n* ± SD% = the number of cells and the percent standard deviation, a qualitative measurement, or the absolute cell count, as the respective study intended. *** Reported qualitatively, rate %, or in µm/day.

## Data Availability

The original data presented in the study are available in Zenodo at doi:10.5281/zenodo.18830374. Access to the deposited files is restricted. The Zenodo record includes a CSV of included studies (title, journal, year, and related metadata) and basic information describing the data extraction; restricted materials are available via the Zenodo access-request process.
